# Supplementary material for: The Effect of Trap Color on Catches of Monochamus galloprovincialis and Three Most Numerous Non-Target Insect Species
Source: Insects. 2022 Feb 22;13(3):220. doi: 10.3390/insects13030220 (PMC8956112; doi:10.3390/insects13030220)
Supplement: Supplementary file 1 [file insects-13-00220-s001.zip › Table S1.pdf]

INSECTS

The effect of trap color on catches of *Monochamus galloprovincialis* and three most numerous non-target insect species

Lidia Sukovata, Aleksander Dziuk, Radosław Plewa and Tomasz Jaworski

Table S1. The numbers of immature and mature *M. galloprovincialis* beetles that responded to each of the compared pairs of colors in three laboratory tests

Test	Codes according to RAL or NCS* color systems	Pair of tested colors	Immature beetles		Mature beetles	
			males	females	males	females
**1**	Pairs of contrasting colors					
	2003/5010	Pastel Orange / Gentian Blue	39/11	41/47	18/12	12/18
	2003/3000	Pastel Orange / Flame Red	14/6	14/6		
	6002/5010	Leaf Green / Gentian Blue	9/13	8/12		
	6002/3000	Leaf Green / Flame Red		6/14		
	8011/3000	Nut Brown / Flame Red	13/17	14/16	14/16	15/15
**2**	Pairs of closely related colors					
	2003/1034	Pastel Orange / Pastel Yellow	15/15	16/14	9/21	10/20
	3015/3000	Light Pink / Flame Red	13/16	15/15	22/8	24/6
	4009/4001	Pastel Violet / Red Lilac	15/15	16/14	16/14	11/19
	5015/5010	Sky Blue / Gentian Blue	20/10	14/16	14/16	14/16
	6002/S 4030-B90G1*	Leaf Green / Cyan Green	15/15	17/13	13/17	10/20
	6025/6011	Fern Green / Reseda Green	12/18	15/15	11/19	14/16
**3**	Colors tested against white (unpainted) coroplast					
	1034	Pastel Yellow	8/22	12/18	9/21	12/18
	2003	Pastel Orange	9/21	6/24	7/23	4/26
	4001	Red Lilac		8/22	11/19	9/21
	S 0520-R90B*	Cyan Blue	11/19	13/17	14/16	17/13
	5010	Gentian Blue		8/22	13/17	4/26
	5015	Sky Blue	9/21	10/20	10/20	10/20
	6002	Leaf Green	5/25	10/20	13/17	7/23
	6011	Reseda Green	14/15	11/19	9/21	8/22
	6025	Fern Green		8/22	8/22	2/28

* NCS—Natural Color System®
